# Integrative metabonomics as potential method for diagnosis of thyroid malignancy

**DOI:** 10.1038/srep14869

**Published:** 2015-10-21

**Authors:** Yuan Tian, Xiu Nie, Shan Xu, Yan Li, Tao Huang, Huiru Tang, Yulan Wang

**Affiliations:** 1 CAS Key Laboratory of Magnetic Resonance in Biological Systems, State Key Laboratory of Magnetic Resonance and Atomic and Molecular Physics, National Centre for Magnetic Resonance in Wuhan, Wuhan Institute of Physics and Mathematics, Wuhan, 430071, P.R. China; 2 Department of Pathology, Union Hospital, Tongji Medical College, Huazhong University of Science and Technology, Wuhan, 430022, P.R. China; 3 Department of Surgery, Union Hospital, Tongji Medical College, Huazhong University of Science and Technology, Wuhan, 430022, P.R. China; 4 State Key Laboratory of Genetic Engineering, Collaborative Innovation Center for Genetics and Development, Metabolomics and Systems Biology Laboratory, School of Life Sciences, Fudan University, Shanghai, 200433, P.R. China; 5 Collaborative Innovation Center for Diagnosis and Treatment of Infectious Diseases, Hangzhou, 310058, P.R. China

## Abstract

Thyroid nodules can be classified into benign and malignant tumors. However, distinguishing between these two types of tumors can be challenging in clinics. Since malignant nodules require surgical intervention whereas asymptomatic benign tumors do not, there is an urgent need for new techniques that enable accurate diagnosis of malignant thyroid nodules. Here, we used ^1^H NMR spectroscopy coupled with pattern recognition techniques to analyze the metabonomes of thyroid tissues and their extracts from thyroid lesion patients (n = 53) and their adjacent healthy thyroid tissues (n = 46). We also measured fatty acid compositions using GC−FID/MS techniques as complementary information. We demonstrate that thyroid lesion tissues can be clearly distinguishable from healthy tissues, and malignant tumors can also be distinguished from the benign tumors based on the metabolic profiles, both with high sensitivity and specificity. In addition, we show that thyroid lesions are accompanied with disturbances of multiple metabolic pathways, including alterations in energy metabolism (glycolysis, lipid and TCA cycle), promotions in protein turnover, nucleotide biosynthesis as well as phosphatidylcholine biosynthesis. These findings provide essential information on the metabolic features of thyroid lesions and demonstrate that metabonomics technology can be potentially useful in the rapid and accurate preoperative diagnosis of malignant thyroid nodules.

Thyroid nodules are abnormal growths that form a mass in the thyroid. It is a very common problem in clinical practice, with an estimated prevalence of ~5% of the adult American population by palpation examination, and up to 50% of adults by autopsy and high-resolution ultrasound examinations^1^,^2^. The incidence rate for thyroid cancer is 1–2 per 100,000 people, which accounts for over 95% of the cancers of the entire endocrine system and 3.8% of total human cancers in the United States in 2015^3^,^4^. Generally, thyroid nodules can be classified into two categories: benign and malignant. Most (>80 %) thyroid nodules are benign, such as nodular goiter (NG), adenomatous goiter (AG), follicular adenoma (FA), Hashimoto’s stuma and cysts. Only 5–15% of the thyroid nodules in clinics are malignant^5^,^6^, which include follicular thyroid carcinomas (FTC), papillary thyroid carcinomas (PTC), medullary thyroid carcinomas (MTC), anaplastic thyroid carcinomas (ATC), thyroid lymphomas and metastatic carcinomas. PTC and FTC are differentiated thyroid carcinomas, which are the most common forms of thyroid cancer^7^.

Prognostic treatment strategies of malignancy are different from those of benign thyroid nodules. Therefore being able to effectively differentiate malignant from benign nodules is an important and challenging issue to clinicians. Fine-needle aspiration biopsy (FNAB) (often under ultrasonographic guidance) is currently the most sensitive and specific preoperative indicator of thyroid malignancy^8^. FNAB is an accurate method for the diagnosis of papillary, medullary, and anaplastic carcinoma, however, holds limited usefulness for distinguishing benign from malignant follicular neoplasms. Moreover, FNAB is limited by indeterminate results and non-diagnostic smears which account for 10–20% of all specimens^8^,^9^. These situations underline the need for further clinical research on new preoperative diagnostic tools.

The use of ancillary techniques including immunohistochemistry and molecular analysis can significantly improve distinction of benign and malignant nodules. Immunohistochemistry was extensively used in the past few years in order to improve the yield of “indeterminate” FNABs. Among the various immunomarkers of malignancy, Hector Battifora mesothelial cell (HBME)-1 and galectin-3 have shown the most consistent results^10^. However, these biomarkers have a low specificity or a poor positive predictive value. Recent investigations have revealed the potential benefits of combined microscopic and molecular analysis of thyroid nodules. The emergence of proteomics and genomics techniques has helped to shed light on other possible thyroid tumor markers. Gnomonic research has found that PTC and MTC are associated with point mutations in *K-RAS* and *BRAF* genes, and that FTC harbors PAX8-PPAR gamma fusions genes^11^,^12^,^13^. Proteomic analysis using 2D-gel and mass spectrometry has found associations of several proteins with thyroid tumor malignancy^11^,^14^,^15^.

Whilst these gene mutations and protein changes can potentially clarify thyroid cancer pathogenesis and determine appropriate molecular targeted therapies, metabolic alterations associated with thyroid malignancy can provide end point metabolic changes, providing complementary information for further understanding the pathogenesis of thyroid malignancies. Metabonomics is a well-established technology for detecting the dynamic and global metabolic alterations of patients in response to diseases and drug interventions^16^,^17^. The combination of ^1^H NMR analysis with multivariate data analysis has been widely employed in characterizing pathogenesis and the progression of a series of cancers such as colorectal cancer^18^, liver cancer^19^, and brain cancer^20^.

More recently, a metabonomics investigation based on LC-MS was employed to detect serum metabolites of PTC and nodular goiters, which found that the major difference between malignant and benign nodules is lipid metabolism^21^. Metabonomics studies have also employed high resolution magic-angle spinning (HRMAS) NMR spectroscopy techniques to differentiate benign and malignant thyroid neoplasms. These studies observed that malignant tumors express higher levels of taurine and lactate, and lower levels of choline, phosphocholine (PC), glycerophosphocholine (GPC), *myo*-inositol and *scyllo*-inositol, as compared with benign tumors^6^,^22^. Although metabolic information of intact tissues can be obtained from HRMAS NMR spectra, some small metabolites that carry important information on the pathogenesis of malignancies cannot be detected due to overlapping with lipid signals that dominate the ^1^H HRMAS NMR spectra of intact tissues. To date, there have been no reports regarding the fatty acid composition of thyroid tissues, despite its importance in understanding the cancer pathogenesis and progression.

In this study, we characterized the metabonomes of human thyroid tissue samples and their extracts using NMR and GC-MS profiles based on the combination of multivariate data analysis. The aims of this study are to provide comprehensive metabolic information on malignant and benign thyroids in order to further the understanding of disease pathogenesis, and to explore the feasibility of metabonomics technology as a useful technique for preoperative diagnosis and prognosis of the thyroid nodules.

## Results

### Histological analysis of the samples

**Figure 1:**
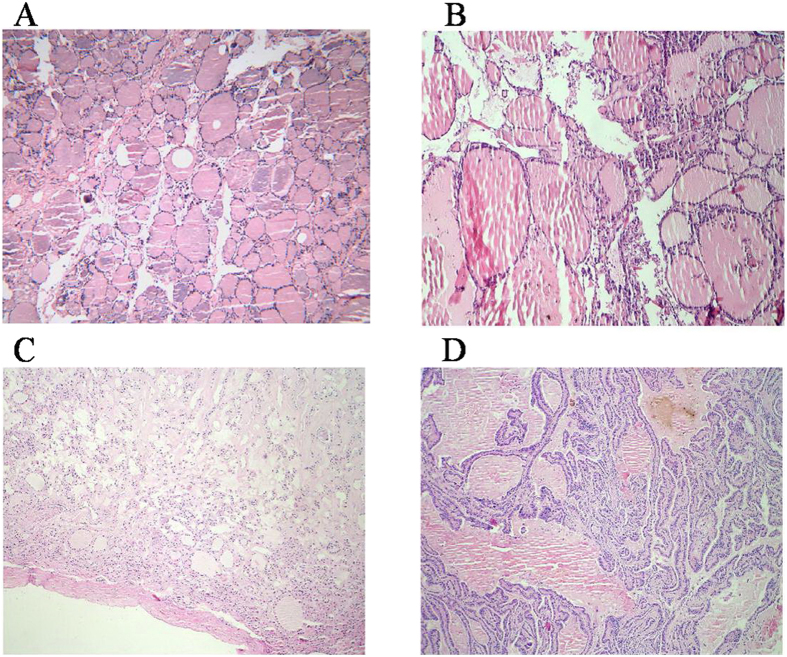
The color photomicrographs of healthy adjacent thyroid tissue (**A**), nodular goiter (**B**), follicular adenoma (**C**) and papillary thyroid carcinoma (**D**).

The healthy adjacent thyroid tissue was composed of colloid-filled follicles, which were lined by regular cuboidal epithelium (Fig. 1A), whereas nodular goiter (NG) consisted of multiple and large dilated follicles lined by flattened epithelium (Fig. 1B). The nodule of follicular adenoma (FA) consisted of uniform compact follicles dissimilar from those of the normal parenchyma (Fig. 1C). The papillary thyroid carcinoma (PTC) was composed of papillae which appeared as fibrovascular stalks lined by the neoplastic epithelial follicular cells (Fig. 1D).

### ^1^H NMR spectroscopy

**Figure 2:**
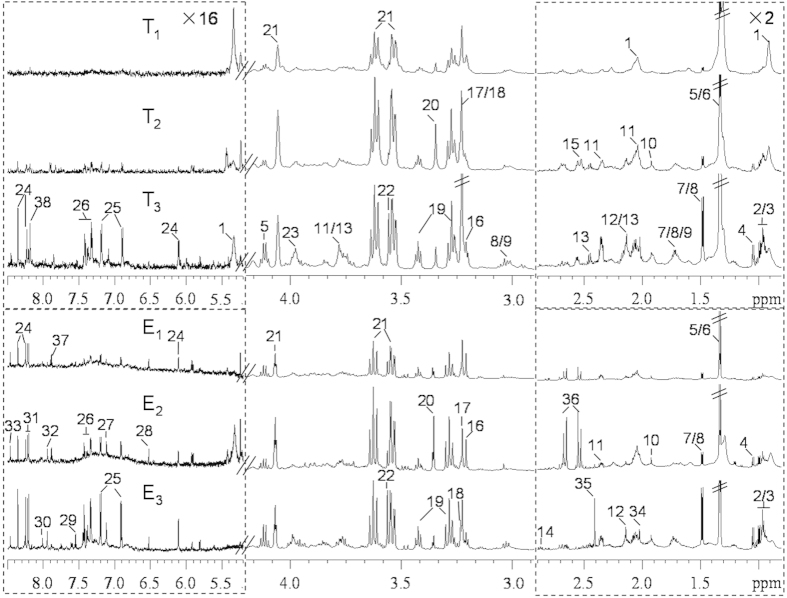
Representative 600 MHz ^1^H spectra of intact thyroid tissue (T) and tissue aqueous extracts (E) originating from healthy adjacent thyroid tissue (T_1_, E_1_), benign thyroid lesion (T_2_, E_2_) and malignant thyroid lesion (T_3_, E_3_). The regions of *δ* 0.8–2.9 and *δ* 5.2–8.5 were vertically expanded 2 and 16 times compared with the region of *δ* 2.9–4.2. Keys: 1, lipid; 2, isoleucine; 3, leucine; 4, valine; 5, lactate; 6, threonine; 7, alanine; 8, lysine; 9, arginine; 10, acetate; 11, glutamate; 12, methionine; 13, glutamine; 14, aspartate; 15, glutathione (GSH); 16, choline; 17, phosphocholine (PC); 18, glycerophosphocholine (GPC); 19, taurine; 20, *scyllo*-inositol; 21, *myo*-inositol; 22, glycine; 23, phosphoethanolamine (PE); 24, inosine; 25, tyrosine; 26, phenylalaine; 27, histidine; 28, fumurate; 29, uracil; 30, guanosine; 31, hypoxanthine; 32, xanthine; 33, formate; 34, acetamide; 35, succinate; 36, citrate; 37, uridine; 38, U1.

The representative ^1^H NMR spectra of tissue extracts and ^1^H HRMAS NMR spectra of intact tissues from healthy thyroid tissue and tissue with lesions illustrated that metabolites in the thyroid tissues mainly included amino acids, the choline-containing compounds, TCA cycle intermediates, lactate, acetamide, acetate, glutathione (GSH), taurine, *myo*-inositol, *scyllo*-inositol, formate, lipid and a number of metabolites of purines and pyrimidines (Fig. 2 and Table S1). These metabolites were assigned based on the previously published data^6^,^23^,^24^ and confirmed by 2D NMR spectra. Clearly, more metabolites were resolved in NMR spectra of tissue extracts than those of intact tissues due to absent signals from lipid resonances in the spectra of tissue extracts. Some obvious differences were also observed between groups. For instance, the lipid levels were lower in the thyroid lesions in comparison with the healthy adjacent thyroid tissue, while the levels of amino acids and choline-containing compounds were higher (Fig. 2).

### Metabolic characteristics of tumor tissues

**Figure 3:**
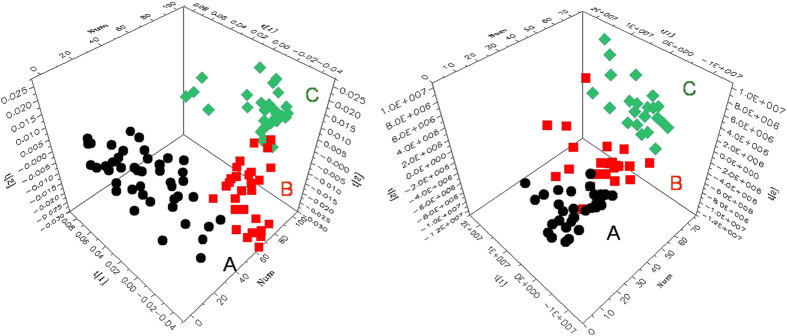
3D PCA scores plots obtained from NMR data of intact thyroid tissues (left) and tissue aqueous extracts (right). (**A**) healthy adjacent thyroid tissues (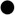), (**B**) benign thyroid lesions (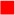), (**C**) malignant thyroid lesions (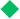). Benign groups: NG and FA. Malignant groups: PTC.

**Figure 4:**
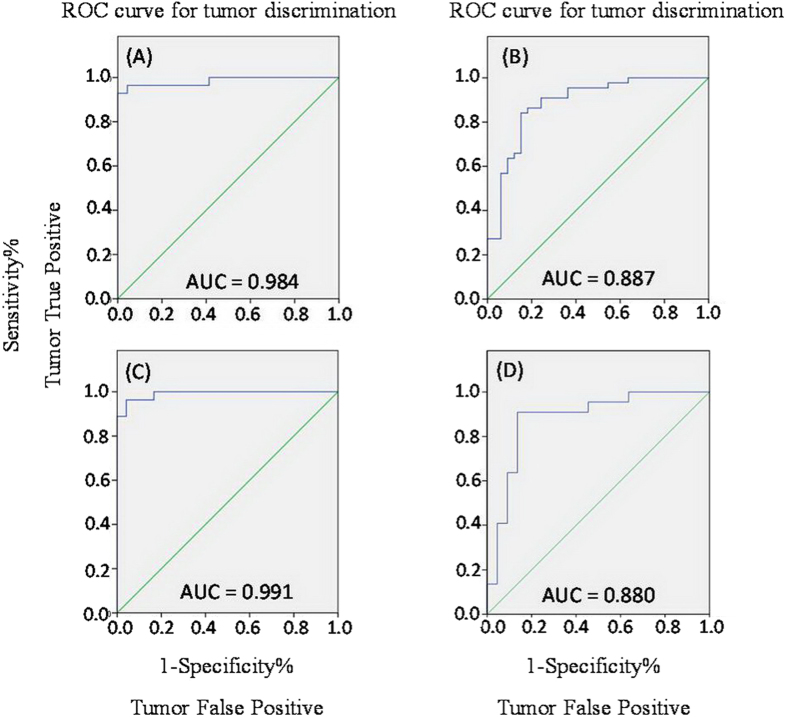
ROC curve obtained from the cross-validated predicted Y-values of the ^1^H NMR OPLS-DA model of intact thyroid tissue (**A,C**) and tissue aqueous extracts (**B,D**), showing the sensitivity and specificity of predictive models in discriminating between thyroid lesions and adjacent healthy thyroid tissue (**A,B**), and between malignant thyroid lesions and benign thyroid lesions (**C,D**).

3D principal components analysis (PCA) results (Fig. 3) showed that, for both intact thyroid tissue and tissue aqueous extracts, separations between the healthy thyroid tissues, benign thyroid lesions and malignant thyroid lesions were present in their respective metabolic profiles. Receiver operating characteristic (ROC) analysis further suggested that thyroid lesions could be confidently distinguished from healthy adjacent tissues and that benign thyroid lesions could be separated from malignant tissues (Fig. 4) using the ^1^H NMR profiles of both intact tissues and their extracts.

**Figure 5:**
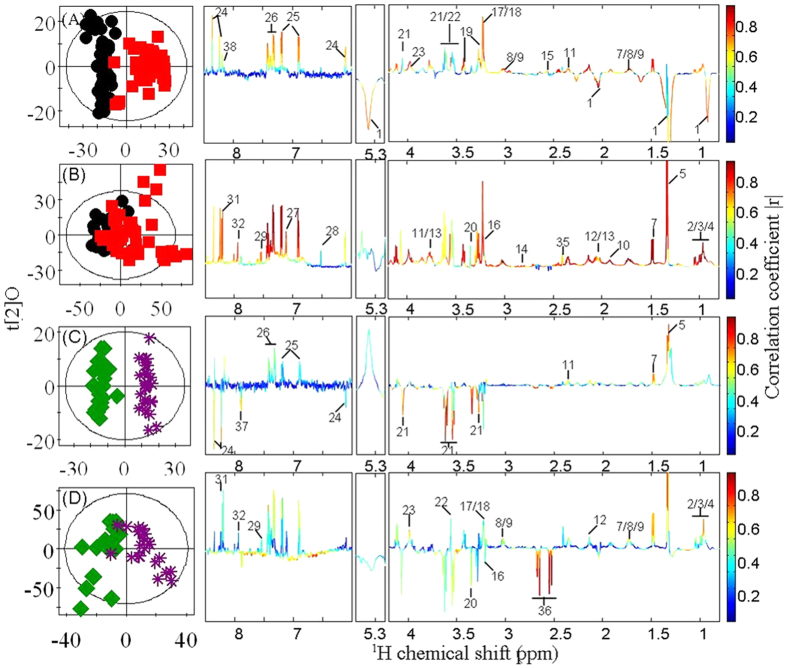
OPLS-DA scores plots (left) and coefficient plots (right) generated from ^1^H NMR spectra of intact thyroid tissue (**A,C**) and tissue aqueous extracts (**B,D**) showing the discrimination between healthy adjacent thyroid tissue (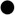), thyroid lesions (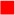), benign thyroid lesions (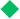) and malignant thyroid lesions (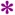). Key to metabolite is given in Table 1.

In order to obtain detailed metabolic changes associated with different tumor groups, we performed orthogonal projection to latent structure-discriminant analysis (OPLS-DA) comparisons between profiles from thyroid lesions and control tissues and between malignant tissues and benign nodules. The model quality (R^2^X and Q^2^, Table S2, Supporting Information) indicated that both benign lesions and malignant tissues could be distinguished from the control samples and benign lesions could be separated from malignant tissues. Further rigorous evaluations with CV-ANOVA (*p* < 0.05) were performed (Table S2, Supporting Information). Compared with the healthy adjacent thyroid tissue, thyroid lesions had significantly higher levels of choline, PC, GPC, phosphoethanolamine (PE), lactate, GSH, taurine, *myo*-inositol, inosine, fumarate and amino acids, and lower levels of lipids (Fig. 5A, Table 1). Due to removal of lipids, additional changes were observed in the tissue extracts of thyroid lesions when compared to healthy adjacent tissues; these included elevations of acetate, succinate, *scyllo*-inositol, formate, and the metabolites from amino acid and nuclear acid metabolism (Fig. 5B, Table 1).

Furthermore, the metabolic differences between benign thyroid tissues and malignant thyroid tissues were clearly visible. The higher levels of PC, GPC, PE, lactate and amino acids but lower levels of citrate, *scyllo*-inositol, *myo*-inositol, inosine and uridine were found in malignant thyroid tissues (Fig. 5C, Table 1). Similar to the metabolic profiles of intact tissues, we also observed significant changes in the tissue extracts. Such changes were highlighted in the additional elevations of uracil, hypoxanthine, xanthine and some amino acids and depleted level of choline when comparing the extracts of malignant thyroid tissues to benign thyroid tissues (Fig. 5D, Table 1).

### GSH and GSSG levels in tumor tissues

**Figure 6:**
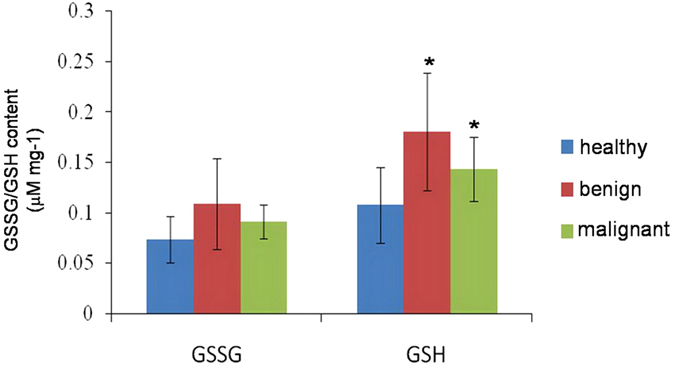
GSSG and GSH contents in healthy adjacent thyroid tissue (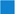), benign thyroid lesions (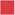) and malignant thyroid lesions (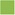). *p < 0.05 when compared to healthy adjacent thyroid tissues.

Analysis of GSH and oxidized glutathione (GSSG) levels confirmed healthy thyroid tissue expressed significantly lower levels of GSH, as compared with benign thyroid lesions and malignant thyroid lesions (Fig. 6). However, no significant difference in the levels of GSH was observed between benign thyroid lesions and malignant thyroid lesions. In addition, there were no significant differences in the levels of GSSG among the three groups of tissues.

### Fatty acid changes in thyroid tissue

**Figure 7:**
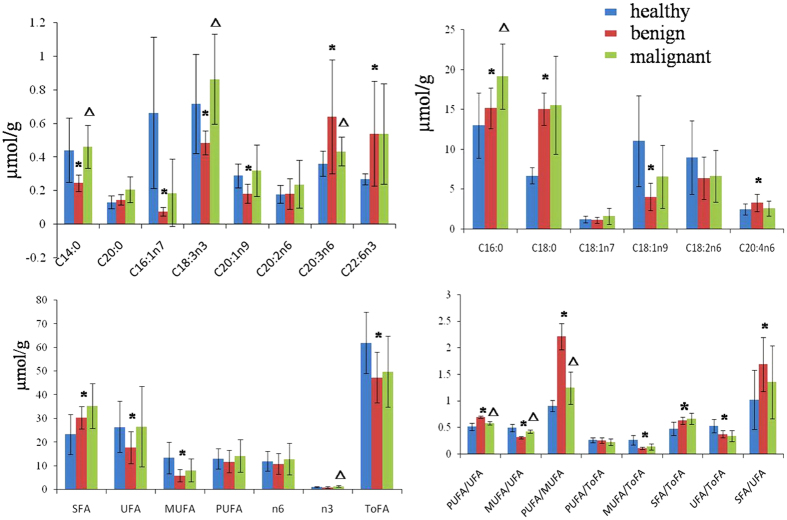
The levels of fatty acids measured by GC-FID/MS. (**A**) healthy adjacent thyroid tissue (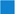), (**B**) benign thyroid lesions (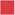) and (**C**) malignant thyroid lesions (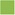). *p < 0.05 when compared to healthy adjacent thyroid tissue, Δ*p* < 0.05 when compared to benign thyroid tissue.

Fatty acid analysis showed remarkable changes in individual fatty acids in the three types of thyroid tissues. As compared with the healthy adjacent thyroid tissue, benign thyroid lesions had significantly lower levels of many fatty acids, such as C14:0, C16:1n7, C18:1n9, C20:1n9 and C18:3n3 but higher levels of C16:0, C18:0, C20:3n6, C20:4n6 and C22:6n3 (Fig. 7 and Table S3). Moreover, the levels of monounsaturated fatty acids (MUFA) and unsaturated fatty acids (UFA) were significantly lower, whereas the saturated fatty acids (SFA) was significantly higher in the benign thyroid lesions compared with the healthy adjacent thyroid tissues. On the other hand, malignant thyroid lesions had significant higher levels of C14:0, C16:0 and C18:3n3 but lower level of C20:3n6 compared with benign thyroid lesions. In addition, the higher levels of delta-6 desaturase (D6D) were also observed in thyroid nodule lesions (Table S3, Supporting Information).

## Discussion

The clinical significance of thyroid nodules, besides the infrequent local compressive symptoms or thyroid dysfunction, is mostly related to the requirement of removing a malignant tumor. This is because malignant thyroid tumors should be controlled aggressively with thyroidectomy, while most benign thyroid tumors need only a conservative management^1^,^25^,^26^. However, the preoperative strategy of differentiating benign thyroid nodules from potential malignant thyroid nodules remains undefined^27^. Recently, studies on the gene-expression of malignant thyroid nodules showed improved separation between benign and malignant thyroid nodules^5^,^28^. Moreover, next-generation sequencing (NGS) of thyroid cancer-related genetic markers (ThyroSeq v2) was applied to screen malignant thyroid nodules using fine-needle aspiration samples and showed high predictive power for follicular neoplasm (FN) and suspicious FN^29^. Here, we used a metabonomics approach to investigate metabolic characteristics associated with various thyroid nodule tissues and tissue extracts. Our results clearly demonstrated that malignant thyroid lesions can be distinguished from the benign thyroid lesions with high specificity and sensitivity, which highlights the importance of the metabonomics as a potentially useful tool for differentiating malignant thyroid nodules from benign tissues and for providing guides for clinical management of thyroid malignancy.

We noted changes in energy metabolism in thyroid lesions. First of all, an increase in anaerobic glycolysis is associated with thyroid lesions, which is manifested in the increased levels of alanine and lactate in thyroid lesions. This observation is consistent with the Warburg Effect, which is a common feature in tumors^30^. The significantly higher concentrations of alanine are also associated with glycolysis mainly via the transamination reaction with pyruvate, which is also consistent with increased glycolytic flux in tumors^31^. High glycolytic flux can also lead to elevations in the TCA cycle; here we observed increased levels of succinate and fumarate in thyroid lesions. Mutations in fumarate hydratase and succinate dehydrogenase have been found previously in human tumors^32^,^33^,^34^, which could explain our observed changes in the levels of TCA cycle intermediates in thyroid lesions. Furthermore, lipid metabolism is also affected by thyroid lesions. Like glucose metabolism, lipid metabolism in tumors is also regulated by the oncogenic signal pathways, which is critical to the initiation and progression of tumors^35^. The increased *de novo* lipogenesis in tumors has been well described^36^,^37^. Interestingly, recent studies also reported that lipolysis and lipid oxidation is also upregulated in tumors^38^,^39^. In our studies, the reductions in net lipid levels were observed, indicating that lipolysis and fatty acid oxidation is overwhelmingly dominating the bioenergetic pathway(s) in thyroid lesions. In addition, we also observed altered fatty acid compositions in the thyroid nodule tissues. For example, a significant increase of saturated fatty acids, palmitate (C16:0) and stearic (C18:0) acid levels were detected in thyroid lesion tissues. SFA has been shown to be capable of repairing DNA damage^40^, hence the presence of high levels of SFA in thyroid lesion tissues is associated with the regulatory system attempting to restore the cancer causing DNA damage. The upregulated activity of D6D was also associated with thyroid nodule lesions. D6D is a rate-limiting enzyme for producing n-6 arachidonic acid^41^ and its upregulation has been previously observed in melanomas and lung tumors^42^. The omega-6 fatty acids are pro-inflammatory molecules and can cause diseases. Recent studies have shown that high levels of pro-inflammatory eicosanoids can accelerate tumor growth and metastasis^43^.

We also observed higher contents of a range of amino acids in the thyroid nodule tissues when compared with the healthy adjacent thyroid tissues. Amino acids in tissues indicates dynamics of protein turn over^44^,^45^,^46^, hence their upregulation in tumor tissues could suggest that high levels of protein turnover helps to maintain the need for tumor growth. High levels of GSH were also noted in the thyroid nodule tissues. This appeared to be a common factor for other tumors, including bone marrow, breast, colon, larynx and lung tumors^47^,^48^. GSH, synthesized from glutamate, cysteine and glycine, plays an anti-oxidative role in many important processes, such as regulating mutagenesis, DNA synthesis, and multidrug and radiation resistance^49^. The increased levels of GSH observed here could be associated with anti-mutagenesis in DNA replication.

We also observed elevations in the levels of nucleic acid, nucleosides and nucleotides in the thyroid nodule lesions. These metabolites are precursors and sources of energy for DNA and RNA biosynthesis, which are highly active during tumor growth. Previous research showed that metabolic changes in nucleotide metabolism occurs during carcinogenesis^49^. For example, PI3K/Akt and Myc pathways are activated in cancers; these two pathways induce diversion of glucose metabolism from glycolytic flux to the pentose phosphate pathway, generating ribose 5-phosphate for *de novo* nucleotides biosynthesis in neoplastic cells^50^. In addition, *Myc*-induced glutamine metabolism also upregulated the activity of genes encoding key enzymes in nucleotides synthesis, such as thymidylate synthase, inosine monophosphate dehydrogenase 1 and 2 and phosphoribosyl pyrophosphate synthetase 2^51^,^52^. Furthermore, reduced activity of dihydropyrimidine dehydrogenase (DPD) is reported to associate with colorectal and hepatocellular carcinoma, which could lead to decrease uracil catabolism^18^,^53^. These evidences together with our observations suggested that alterations of nucleotide metabolism are associated with thyroid lesions.

Most importantly, we noted elevations in the levels of choline-containing compounds, such as choline, PC, GPC and PE in the thyroid nodule tissues, which is consistent with previous observations made in other malignant tumors^53^. The activation of several enzymes involved in phosphatidylcholine biosynthesis has been observed in ovarian carcinoma^18^,^54^ and could contribute to observed elevations in the levels of choline-containing compounds. The elevated levels of choline-containing compounds could be used for increased cellular proliferation and membrane biosynthesis in thyroid nodule tissues. In addition, high levels of taurine, *myo*-inositol and *scyllo*-inositol were also observed in the thyroid nodule tissues, which are in good agreement with what has been reported previously for colorectal tumor and squamous cell carcinoma^18^,^54^. The elevations in these osmolytes may indicate localized changes in osmoregulation and volume regulation of tumor.

In a summary, this study has shown the feasibility of integrative metabonomics (combining NMR spectroscopy and GC-MS) in distinguishing malignant thyroid lesions from benign thyroid lesions. The pathogenesis and progression of thyroid nodules were accompanied with multiple metabolic pathways including glycolysis, TCA cycle, and metabolisms of lipids, fatty acids, amino acids, choline metabolism and nucleotide biosynthesis. This metabolic information can help to further our understanding of the pathogenesis of thyroid lesions. Although the method showed potential application in clinical management of thyroid nodules, an independent validation including a lager replication cohort is necessary to further validate the model before applying in clinics.

## Materials and Methods

### Ethics statement

The methods used were carried out in accordance with the approved guidelines. All experimental protocols were approved by Wuhan Institute of Physics and Mathematics, University of Chinese Academy of Sciences.

### Chemicals

Deuterium oxide (D_2_O, 99.9% D) and sodium 3-(trimethylsilyl) [2,2,3,3-^2^H_4_] propionate (TSP) were purchased from Cambridge Isotope Laboratories, Inc. (Miami, U.S.A.). Methanol, hexane, K_2_CO_3_, K_2_HPO_4_·3H_2_O, and NaH_2_PO_4_·2H_2_O (all in analytical grade) were obtained from Sinopharm Chemical Reagent Co. Ltd. (Shanghai, China). Methyl heptadecanoate, methyl tricosanate, and acetyl chloride (99.0%) were bought from Sigma-Aldrich (St. Louis, MO). 3,5-Di-tert-butyl-4-hydroxytoluene (BHT) and a mixed standard methyl esters of 37 fatty acids were obtained from Supelco (Bellefonte, PA). A buffer containing 0.001% TSP and 80% D_2_O was prepared with K_2_HPO_4_ and NaH_2_PO_4_ (0.15 M, pH 7.43) and used as solvent for NMR analysis of tissue extracts.

### Clinical population and sample collection

Human thyroid lesions and comparative healthy thyroid tissues were collected from 53 patients at the Department of Pathology and General Surgery, Union Hospital, Tongji College of Medicine, Huazhong University of Science and Technology. The clinicopathological characteristics of the patients are summarized in Table 2. Matched nodule tissue and healthy adjacent thyroid samples were obtained from 46 out of 53 patients. Only nodule tissues of the remaining 7 patients were excised. All samples were frozen in liquid nitrogen, then stored at −80 °C until further processing. The study protocol was approved by the ethics committee and the informed consents were received from all patients.

### Histopathological assessment

Both the excised nodule specimens and the healthy adjacent thyroid tissues used for spectroscopy experiments were separately assessed by histopathology. The fomalin-fixed samples were sectioned and stained using Hematoxylin and Eosin (H&E) methods. The microscopic assessments were performed and evaluated by pathologists microscopically.

### Evaluation of GSH and GSSG

Both GSH and GSSG were measured using a GSH and GSSG Assay Kit (Shanghai, China). The results were analyzed using Student’s t-test to test statistical significance. A probability of less than 5% (*p* < 0.05) was considered significant.

### Sample preparation for NMR spectroscopy

Tissue samples (about 10 mg) were individually placed in D_2_O saline and inserted into a 4 mm diameter zirconium oxide rotor for the HRMAS NMR spectral acquisition.

Parallel tissue samples (about 50 mg) from patients were extracted three times using a tissuelyzer (QIAGEN TissueLyserII, Germany) with methanol-water mixture (2:1, v/v). Three supernatants obtained were pooled together and freeze-dried after removal of methanol *in vacuo*. The solid residues were weighed and individually dissolved into 550 μL phosphate buffer. After centrifugation (16099 g, 4 °C) for 10 min, 500 μL of such solution of supernatant was transferred into 5 mm NMR tubes.

### ^1^H NMR spectroscopic analysis

All ^1^H HRMAS NMR spectra were obtained at 283 K on a Bruker AVIII NMR spectrometer (Bruker Biospin, Germany) equipped with a HRMAS probe with a spin rate of 5000 Hz. In order to attenuate NMR signals of macromolecules, Carr-Purcell-Meiboom-Gil (CPMG) spectrum was collected for each tissue. The spin-spin relaxation delay, 2nτ, was set to 70 ms for all tissue samples. ^1^H NMR spectra of tissue extracts were operated at 600.13 MHz for ^1^H and recorded at 298 K on a Bruker Avance III 600 MHz NMR spectrometer configured inverse cryogenic probe. The first increment of NOESY pulse sequence [RD-90°-t1-90°-tm-90°-ACQ] were recorded for tissue extracts with a recycle delay (2 s) and mixing time (tm, 80 ms). Typically, the 90° pulse length was about 10 μs and 128 transients were recorded into 32 k data points with a spectral width of 20 ppm.

To facilitate NMR signal assignments, 2D NMR spectra including ^1^H−^1^H correlation spectroscopy (COSY), ^1^H−^1^H total correlation spectroscopy (TOCSY), ^1^H J-resolved spectroscopy (JRES), ^1^H−^13^C heteronuclear single-quantum correlation spectroscopy (HSQC), and ^1^H−^13^C heteronuclear multiple bond correlation spectroscopy (HMBC) were acquired for selected samples and processed as previously described^55^,^56^.

### Data processing and multivariate data analysis

Free induction decays were multiplied by an exponential window function with a 1.0 Hz line broadening factor prior to Fourier transformation. All spectra were phase- and baseline-corrected, and referenced internally to the β protons of alanine (*δ* = 1.48) for intact tissue and TSP (*δ* = 0.00) for tissue extracts using Topspin (V3.0, Bruker Biospin, Germany). The regions of *δ* 0.5–9.0 were divided into 0.004 ppm-width buckets using AMIX software package (V3.9.5, Bruker Biospin, Germany). In order to remove the effect of imperfect water saturation, the regions in the range *δ* 4.20–5.20 were discarded prior to analysis. The buckets were normalized to the sum of total integrals for intact tissue and wet weight for tissue extracts.

Multivariate data analysis was carried out using software package SIMCA-P^+^ (V12.0, Umea, Sweden). PCA was carried out with mean-center scaling to examine group clustering and detect potential outliers. The OPLS-DA was further constructed using the unit-variance scaling NMR data as the X-matrix and group information as the Y-matrix^57^. The 7-fold cross-validation method^57^ OPLS-DA model were validated. These models were further validated with CV-ANOVA approach (*p* < 0.05)^58^. The back transformed^59^ loadings with color-scaled correlation coefficients of metabolites were plotted in MATLAB using an in-house developed Matlab script (V7.0, the Mathworks Inc., U.S.A.). In these loading plots, the color-coded correlation coefficient showed the variables contributed to the intergroup differentiation, with “warm” color (e.g., red) metabolites reflecting more significant contribution than “cold” color (e.g., blue) ones.

### Receiver Operating Characteristic (ROC) Curve

Receiver operating characteristic (ROC) curve was obtained from Y predicted values to evaluate the predicative ability of OPLS-DA models. Area under the ROC curve (AUC) was computed using the performance curve algorithm from SPSS 18.0 (SPSS Inc., Chicago, IL, USA).

### GC-FID/MS analysis of fatty acid composition in thyroid tissue

Quantitative measurements of tissue fatty acids were conducted with a previously reported method^60^. In brief, about 15 mg of tissue sample was homogenized three times in methanol using a TissueLyser (20 Hz, 90 s). After acetyl chloride catalyzed methyl esterification^60^, the identification and quantification of methylated fatty acids was performed on a Shimadzu GCMS-QP2010Plus spectrometer (Shimadzu Scientific Instruments, USA) configured flame ionization detector (FID) and a mass spectrometer by comparing to the internal standards. A set of DB-225 capillary GC column (0.1 μm film thickness, 0.1 mm ID, 10 m) from Agilent Technologies was used. Sample injection volume was 1 μL with a splitter (1:60). The injection port and detector temperature were both set at 230 °C. The MS spectra were acquired with the EI (70 eV) source and a m/z range of 45–450. The fatty acid compositions were calculated as μmol of fatty acids per gram for tissue. The molar percentages were calculated for n3 and n6 type fatty acids, SFA, UFA, MUFA, and polyunsaturated fatty acids (PUFA), respectively.

## Additional Information

**How to cite this article**: Tian, Y. *et al*. Integrative metabonomics as potential method for diagnosis of thyroid malignancy. *Sci. Rep*. **5**, 14869; doi: 10.1038/srep14869 (2015).

## Supplementary Material

Supplementary Information

## Figures and Tables

**Table 1:** Key observed metabolic differences between the healthy adjacent thyroid and benign thyroid lesions, and between benign and malignant thyroid lesions.

metabolite (no.)	intact thyroid tissue		tissue aqueous extracts	
	Node *vs* Healthy n = 44, |*r*| > 0.29	Malignant *vs* Benign n = 25, |*r*| > 0.39	Node *vs* Healthy n = 44, |*r*| > 0.29	Malignant *vs* Benign n = 25, |*r*| > 0.39
Lipid (1)	−0.65	—	—	—
Citrate (36)	—	−0.59	—	−0.79
Isoleucine (2)	0.65	0.41	0.97	0.57
Leucine (3)	0.67	0.44	0.96	0.51
Valine (4)	0.71	0.47	0.95	0.51
Alanine (7)	0.75	0.66	0.95	0.67
Lysine (8)	0.75	0.49	0.93	0.54
Arginine (9)	0.73	0.49	0.93	0.54
Glutamate (11)	0.69	0.60	0.91	0.45
Methionine (12)	0.85	0.62	0.96	0.51
Glutamine (13)	0.82	—	0.82	—
Aspartate (14)	—	—	0.84	—
Glycine (22)	0.78	—	0.90	0.46
Tyrosine (25)	0.73	—	0.97	0.57
Phenylalanine (26)	0.66	0.51	0.95	0.60
Histidine (27)	—	—	0.92	—
Choline (16)	0.63	—	0.84	−0.39
PC (17)	0.79	0.55	0.97	0.50
GPC (18)	0.79	0.55	0.97	0.50
PE (23)	0.76	0.49	0.85	0.60
Lactate (5)	0.32	0.71	0.91	0.67
Acetate (10)	—	—	0.95	—
Succinate (35)	—	—	0.55	—
GSH (15)	0.48	—	—	—
Taurine (19)	0.83	—	0.91	—
*Scyllo*-inositol (20)	—	−0.66	0.55	−0.49
*Myo*-inositol (21)	0.44	−0.87	0.59	−0.46
Inosine (24)	0.65	−0.49	0.67	—
Uracil (29)	—	—	0.88	0.42
Hypoxanthine (31)	—	—	0.82	0.55
Xanthine (32)	—	—	0.83	0.45
Uridine (37)	—	−0.74	0.65	−0.56
Fumarate (28)	0.29	—	0.50	—
Formate (33)	—	—	0.67	—
U1 (38)	0.59	—	—	—

The coefficients from OPLS-DA results; positive and negative signs indicate positive and negative correlation in the concentrations, respectively.

**Table 2:** Clinical features of patients in this study^a^.

	patients for HRMAS NMR	patients for tissue extract
Number	53	50
Age (median, range)	46, 21–75	44, 21–71
Male/female ratio	9/44	7/43
NG	22	22
FA	3	3
PTC	28	25

^a^NG, nodular goiter; FA, follicular adenoma; PTC, papillary thyroid carcinoma.
