# Supplementary material for: Integrative metabonomics as potential method for diagnosis of thyroid malignancy
Source: Sci Rep. 2015 Oct 21;5:14869. doi: 10.1038/srep14869 (PMC4613561; doi:10.1038/srep14869)
Supplement: Supplementary Information [file srep14869-s1.pdf]

Integrative metabonomics as potential method for diagnosis of thyroid malignancy

Yuan Tian[†ζ], Xiu Nie[‡ζ], Shan Xu[†], Yan Li[‡], Tao Huang[‡*], Huiru Tang[Φ*], Yulan Wang[†#*]

[†]CAS Key Laboratory of Magnetic Resonance in Biological Systems, State Key Laboratory of Magnetic Resonance and Atomic and Molecular Physics, National Centre for Magnetic Resonance in Wuhan, Wuhan Institute of Physics and Mathematics, University of Chinese Academy of Sciences, Wuhan, 430071, P. R. China

[‡]Department of Pathology, Union Hospital, Tongji Medical College, Huazhong University of Science and Technology, Wuhan, 430022, P.R. China

[Φ]State Key Laboratory of Genetic Engineering, Collaborative Innovation Center for Genetics and Development, Metabolomics and Systems Biology Laboratory, School of Life Sciences, Fudan University, Shanghai, 200433, P. R. China.

[#]Collaborative Innovation Center for Diagnosis and Treatment of Infectious Diseases, Hangzhou, 310058, P. R. China.

Supplementary Files

Table S1. NMR assignment for the metabolites found in thyroid tissues

no.	metabolites	moieties	δ ^{1}H (ppm) and multiplicity[a]	δ ^{13}C (ppm)
1	lipid	$CH_3(CH_2)_n$	0.86(t)	15.0
		$CH_3CH_2CH_2C=$	0.88(t)	15.0
		$(CH_2)_n$	1.27(m)	32.0
		CH_2CH_2CO	1.57(m)	27.5
		$CH_2C=C$	2.01(m)	29.5
		CH_2CO	2.23(m)	36.0
		$C=CCH_2C=C$	2.76(m)	28.5
		-CH=CH-	5.30(m)	130.0
2	isoleucine	δCH_3	0.94(t)	14.2
		γCH_3	1.02(d)	17.2
		γCH_2	1.26(m)	27.5
		$\gamma' CH_2$	1.48(m)	27.5
		βCH	1.98(m)	37.7
		αCH	3.67(d)	62.4
3	leucine	δCH_3	0.96(d)	24.5

		δCH_3	0.97(d)	24.2
		γCH	1.72(m)	27.0
		βCH_2	1.72(m)	42.6
		αCH	3.74(t)	57.2
4	valine	γCH_3	0.99(d)	19.6
		γCH_3	1.04(d)	20.8
		βCH	2.27(m)	32.6
		αCH	3.62(d)	63.3
5	lactate	CH_3	1.33(d)	22.9
		CH	4.11(q)	71.3
6	threonine	γCH_3	1.33(d)	22.5
		αCH	3.59(d)	63.1
		βCH	4.26(m)	69.0
7	alanine	βCH_3	1.48(d)	19.0
		αCH	3.79(q)	53.4
8	lysine	γCH_2	1.48(m)	24.1
		δCH_2	1.72(m)	29.2
		βCH_2	1.90(m)	33.0
		εCH_2	3.03(t)	42.2
		αCH	3.76(t)	57.6
9	arginine	γCH_2	1.73(m)	27.0
		βCH_2	1.93(m)	30.6
		δCH_2	3.23(t)	43.4
		αCH	3.75(t)	57.6
		C=NH		159.6
10	acetate	CH_3	1.92(s)	26.2
11	glutamate	βCH_2	2.09(m)	29.9
		$\beta' CH_2$	2.10(m)	29.9
		γCH_2	2.36(m)	36.4
		αCH	3.77(m)	57.2
12	methionine	δCH_3	2.14(s)	16.8
		βCH_2	2.16(m)	33.0
		γCH_2	2.65(t)	31.7
		αCH	3.86(m)	56.9
13	glutamine	βCH_2	2.10(m)	30.0
		γCH_2	2.46(m)	33.8
		αCH	3.77(m)	57.5
14	aspartate	βCH_2	2.68(m)	39.5
		$\beta' CH_2$	2.82(m)	39.8
		αCH	3.91(m)	55.3
15	glutathione(GSH)	$3CH_2$	2.17(m)	#
		$4CH_2$	2.56(m)	#
		$12CH_2$	2.94(m)	#
		$12'CH_2$	2.97(m)	#

		2CH	3.79(t)	#
		7CH	4.56(t)	#
16	choline	$N(CH_3)_3$	3.21(s)	56.8
		NCH_2	3.52(m)	74.1
		OCH_2	4.07(m)	54.1
17	phosphocholine(PC)	$N(CH_3)_3$	3.23(s)	57.0
		NCH_2	3.61(m)	69.3
		OCH_2	4.17(m)	58.0
18	glycerophosphocholine(GPC)	$N(CH_3)_3$	3.23(s)	57.0
		NCH_2	3.69(m)	69.0
		OCH_2	4.34(m)	62.7
19	taurine	CH_2SO_3	3.28(t)	50.7
		NCH_2	3.43(t)	38.2
20	*scyllo*-inositol	CH_3	3.35(s)	76.6
21	*myo*-inositol	5CH	3.27(t)	77.2
		1CH,3CH	3.54(m)	74.2
		4CH,6CH	3.63(m)	75.3
		2CH	4.07(m)	75.1
22	glycine	CH_2	3.56(s)	44.3
23	phosphoethanolamine(PE)	CH_2NH_3	3.23(t)	43.8
		CH_2OH	3.99(m)	63.1
24	inosine	CH_2	3.85(dd)	63.8
		$'CH_2$	3.92(dd)	63.8
		5H'	4.28(q)	88.6
		4H'	4.44(t)	73.4
		2H'	6.11(d)	91.1
		8H	8.25(s)	149.8
		2H	8.36(s)	143.2
25	tyrosine	βCH_2	3.06(dd)	38.3
		$\beta' CH_2$	3.15(dd)	38.3
		αCH	3.94(dd)	59.2
		3 or 5CH	6.91(d)	118.9
		2 or 6CH	7.20(d)	133.8
26	phenylalanine	βCH_2	3.13(dd)	38.4
		$\beta' CH_2$	3.29(dd)	38.4
		αCH	3.98(dd)	59.3
		2 or 6CH	7.33(m)	132.1
		4CH	7.38(m)	131.2
		3 or 5CH	7.43(m)	132.2
27	histidine	5CH	7.09(s)	120.1
		3CH	7.83(s)	138.3
28	fumarate	CH	6.52(s)	138.5
29	uracil	CH	5.81(d)	100.9
		CH	7.54(d)	146.5

30	guanosine	CH	8.01(s)	#
31	hypoxanthine	2CH	8.20(s)	148.6
		7CH	8.22(s)	145.0
32	xanthine	CH	7.94(s)	139.1
33	formate	CH	8.45(s)	#
34	acetamide	CH_3	2.02(s)	24.7
35	succinate	CH_2	2.41(s)	37.0
36	citrate	CH_2	2.54(d)	46.5
		$'CH_2$	2.66(d)	46.5
37	uridine	$'CH_2$	3.92(d)	64.3
		4H'	4.14(dd)	86.6
		3H'	4.24(dd)	73.1
		2H'	4.36(dd)	78.0
		5H	5.91(d)	95.0
		6H	5.92(d)	90.5
		1H'	7.89(d)	144.0
38	U1		8.19(s)	#

Table S2. Summary of OPLS-DA models[a].

	intact thyroid tissue				tissue aqueous extracts			
	A vs. (B+C)	A vs. B	B vs. C	A vs. C	A vs. (B+C)	A vs. B	B vs. C	A vs. C
R^2X	0.28	0.33	0.16	0.35	0.78	0.82	0.77	0.73
Q^2	0.75	0.81	0.77	0.77	0.33	0.31	0.44	0.49
p	3.9×10^{-25}	1.5×10^{-22}	4.7×10^{-14}	1.1×10^{-20}	8.1×10^{-6}	1.0×10^{-3}	1.3×10^{-4}	7.3×10^{-7}

[a]A: healthy adjacent thyroid tissue, B: benign thyroid lesions, C: malignant thyroid lesions.

Table S3. Fatty acid compositions in thyroid tissues.

	**Healthy**[a] (*n* = 25)	**Benign** (*n* = 25)	**Malignant** (*n* = 15)	***p* values** (healthy *vs* benign)	***p* values** (healthy *vs* malignant)	***p* values** (benign *vs* malignant)
SFA[b]	23.343±8.423	30.412±4.751	35.377±9.423	1.63E-2	2.02E-2	3.08E-1
C14:0	0.443±0.191	0.247±0.049	0.462±0.127	4.48E-3	8.43E-1	1.82E-2
C16:0	13.005±4.115	15.193±2.558	19.159±4.081	4.94E-2	1.30E-2	6.93E-3
C18:0	6.681±1.029	15.083±2.020	15.550±6.164	1.21E-12	4.89E-3	8.75E-1
C20:0	0.133±0.039	0.146±0.031	0.207±0.078	2.51E-1	1.00E-1	1.59E-1
UFA[b]	26.445±10.818	17.739±6.759	26.651±17.083	3.66E-3	9.76E-1	3.11E-1
MUFA[b]	13.415±6.655	5.913±2.558	8.210±4.780	2.45E-3	1.74E-1	4.11E-1
C16:1n7	0.665±0.452	0.075±0.026	0.187±0.200	8.61E-4	6.35E-2	3.46E-1
C18:1n7	1.186±0.411	1.112±0.385	1.599±1.031	6.15E-1	4.31E-1	3.53E-1
C18:1n9	11.031±5.679	4.024±1.698	6.568±3.960	1.28E-3	1.71E-1	2.90E-1
C20:1n9	0.291±0.071	0.184±0.056	0.320±0.152	1.26E-5	6.00E-1	1.73E-1
PUFA[b]	13.030±4.360	11.827±4.709	14.240±6.921	4.54E-1	6.66E-1	3.33E-1
n6 [b]	11.979±4.145	10.832±4.449	12.834±6.647	4.51E-1	7.49E-1	3.96E-1
C18:2n6	8.967±4.651	6.372±2.654	6.621±3.226	9.15E-2	3.70E-1	8.65E-1
C20:2n6	0.180±0.052	0.182±0.090	0.239±0.143	9.49E-1	4.75E-1	2.84E-1
C20:3n6	0.361±0.075	0.642±0.339	0.436±0.086	2.97E-4	1.23E-1	1.57E-2
C20:4n6	2.446±0.721	3.267±1.106	2.575±0.930	2.61E-2	7.60E-1	2.04E-1
n3[b]	1.051±0.306	0.995±0.366	1.406±0.402	6.43E-1	6.52E-2	2.97E-2
C18:3n3	0.719±0.295	0.486±0.070	0.865±0.267	2.01E-2	3.54E-1	3.32E-2
C22:6n3	0.269±0.034	0.539±0.312	0.540±0.299	1.04E-4	1.12E-1	9.94E-1
ToFA[b]	62.029±12.976	47.414±10.629	49.788±14.968	1.20E-2	4.63E-2	5.48E-1
n6/n3[b]	11.663±3.017	9.874±0.630	7.689±2.052	6.66E-1	2.94E-2	1.22E-1
PUFA/UFA[b]	0.509±0.070	0.690±0.020	0.579±0.028	1.47E-6	1.22E-1	1.35E-8
MUFA/UFA[b]	0.491±0.070	0.310±0.020	0.421±0.028	1.47E-6	1.22E-1	1.35E-8
PUFA/MUFA[b]	0.904±0.109	2.214±0.246	1.244±0.306	3.84E-19	1.08E-2	2.20E-07
PUFA/ToFA[b]	0.262±0.047	0.252±0.051	0.221±0.066	5.70E-1	1.66E-1	2.46E-1
MUFA/ToFA[b]	0.264±0.089	0.109±0.019	0.136±0.058	7.90E-5	1.86E-2	4.28E-1
SFA/ToFA[b]	0.473±0.127	0.628±0.068	0.665±0.107	1.41E-3	1.72E-2	3.56E-1
UFA/ToFA[b]	0.527±0.127	0.372±0.068	0.335±0.107	1.41E-3	1.72E-2	3.56E-1
SFA/UFA[b]	1.020±0.559	1.687±0.506	1.351±0.683	8.26E-4	3.47E-1	2.46E-1
SCD16[c]	0.053±0.035	0.005±0.001	0.009±0.009	5.13E-4	1.26E-3	3.69E-1
SCD18[c]	1.459±1.033	0.255±0.090	0.372±0.203	1.96E-3	4.29E-3	3.34E-1
D5D[c]	5.817±0.834	5.466±1.193	4.835±0.865	4.04E-1	5.33E-2	2.75E-1
D6D[c]	0.058±0.048	0.098±0.034	0.081±0.045	2.00E-3	2.78E-1	3.28E-1

[a] data are presented as mean ± SD (μmol/g)

[b] SFA: saturated fatty acids; UFA: unsaturated fatty acids; MUFA: monounsaturated fatty acids; PUFA: polyunsaturated fatty acids; n6: n6 PUFA ; n3: n3 PUFA ;ToFA: total fatty acids; n6/n3: n6-to-n3 ratio; PUFA/UFA:

PUFA-to-UFA ratio; MUFA/UFA: MUFA-to-UFA ratio; PUFA/MUFA: PUFA-to-MUFA ratio; PUFA/ToFA: PUFA-to-ToFA ratio; MUFA/ToFA: MUFA-to-ToFA ratio; SFA/ToFA: SFA-to-ToFA ratio; UFA/ToFA: UFA-to-ToFA ratio; SFA/UFA: SFA-to-UFA ratio; SFA = C14:0+C16:0+C18:0+C20:0; UFA = MUFA+PUFA; MUFA = C16:1n7+C18:1n7+C18:1n9+C20:1n9; PUFA = n6+n3; n6 = C18:2n6+C18:3n6+C20:2n6+C20:3n6+C20:4n6 ; n3 = C18:3n3+C20:5n3+C22:6n3

[c] SCD16: stearoyl-CoA desaturase-16; SCD18: stearoyl-CoA desaturase-18; D5D: Δ5-desaturase; D6D: Δ6-desaturase. Hepatic desaturase were calculated by product-precursor ratio. SCD16 = C16:1n7/C16:0; SCD18 = C18:1n9/C18:0 ; D5D = C20:4n6/C20:3n6 ; D6D = C20:3n6/C18:2n6
